# Association between copper exposure and renal fibrosis in patients with chronic kidney disease: evidence from Mendelian randomization and a retrospective study

**DOI:** 10.3389/fpubh.2025.1657180

**Published:** 2025-08-25

**Authors:** Kaixiang Liu, Min Yu, Yangyang He, Ting Wang, Honghua Hu, Zhengwei Wan, Ping Shuai, Shasha Chen, Guisen Li, Li Wang, Xiang Zhong

**Affiliations:** ^1^Department of Nephrology and Institute of Nephrology, Sichuan Provincial People’s Hospital, School of Medicine, University of Electronic Science and Technology of China, Chengdu, China; ^2^Department of Clinical Laboratory, Sichuan Provincial People’s Hospital, School of Medicine, University of Electronic Science and Technology of China, Chengdu, China; ^3^Department of Health Management Center, Sichuan Provincial People’s Hospital, Institute of Health Management, University of Electronic Science and Technology of China, Chengdu, China

**Keywords:** serum copper, chronic kidney disease, renal fibrosis, Mendelian randomization analysis, retrospective study

## Abstract

**Background:**

Chronic kidney disease (CKD), a global health challenge, is closely linked to renal fibrosis progression. Copper, an essential trace element, influences cellular functions, yet its role in CKD-related fibrosis remains unclear. This study explores the causal relationship between serum copper levels and renal fibrosis in CKD.

**Methods:**

A two-sample Mendelian Randomization (MR) analysis integrated GWAS and FinnGen data. Serum copper and other metals were quantified via ICP-MS in 505 CKD patients and 50 controls. Renal fibrosis was histologically assessed in 168 biopsy-confirmed cases. Multivariable logistic regression and restricted cubic splines (RCS) evaluated associations between copper levels, renal function, and fibrosis severity, adjusting for demographics and biochemical parameters.

**Results:**

MR confirmed causality between elevated copper and CKD risk. CKD patients had higher serum copper than controls (957.10 ± 273.82 vs. 795.50 ± 143.85 ng/ml, *p* < 0.001), with progressive increases from stage 1 to 5 (*p* < 0.001). In biopsy-proven cases, severe fibrosis (>5%) correlated with higher copper levels and lower eGFR versus mild fibrosis (≤5%). Adjusted analysis identified quartile 4 copper levels (>961.64 ng/ml) as an independent predictor of severe fibrosis (OR = 2.75, 95% CI: 1.06–7.16, *p* < 0.001). RCS revealed non-linear relationships between copper, fibrosis (P for non-linear = 0.038), and eGFR (P for non-linear = 0.005).

**Conclusion:**

Elevated serum copper is independently associated with renal fibrosis in CKD, suggesting copper dysregulation may contribute to fibrotic pathogenesis. These findings underscore the therapeutic potential of targeting copper metabolism to mitigate CKD progression.

## 1 Introduction

Chronic kidney disease (CKD) affects over 850 million individuals globally and is projected to become the fifth leading cause of premature mortality worldwide by 2040. Characterized by high prevalence and poor prognosis, CKD significantly increases patient mortality risk and possesses a substantial burden on healthcare systems due to extensive resource utilization, thereby representing a critical public health challenge (1–3). Renal fibrosis is a key pathological characteristic of CKD and leads to irreversible structural damage and progression to end-stage renal disease (ESRD) (4, 5). Therefore, understanding and identifying modifiable risk factors associated with renal fibrosis is critical for developing strategies to delay CKD progression.

Trace elements, including copper, have recently attracted attention for their potential roles in CKD pathogenesis. Copper is an essential trace element involved in various biological processes, such as enzymatic reactions, antioxidant defense, iron metabolism, and immune function (6). However, dysregulation of copper homeostasis can lead to oxidative stress, which con-tributing to renal damage and fibrosis (7). Elevated copper levels promote the generation of reactive oxygen species (ROS), leading to cellular damage in renal tubular cells and podocytes, which accelerates CKD pro-gression (8, 9). Those findings provide evidences supporting the role of copper in the progression of CKD. Those findings provide evidences supporting the role of copper in the progression of CKD.

Several cross-sectional studies have confirmed that high copper exposure is strongly associated with the development and progression of CKD. For instance, a study conducted in Zhejiang Province, China, revealed a dose-dependent positive correlation between blood copper levels and CKD prevalence among older adult participants (10). Similarly, another cross-sectional survey conducted in 2020–2021 among 2,210 adults across 12 Chinese provinces revealed a correlation between urinary metals and metalloids and renal dysfunction, with copper identified as a potential risk factor (11). Studies in some special population, solar greenhouse workers and rural residents, suggested there is a negative association between urinary copper levels and estimated glomerular filtration rate (eGFR) (12, 13). Moreover, CKD patients with prolonged exposure to environmental heavy metals tend to progress rapidly to ESRD (14). Those findings provide additional evidence supporting the role of copper in the progression of CKD. Thus, maintaining optimal copper levels could potentially mitigate renal injury and enhance kidney function in CKD patients.

Additionally, a data from the National Health and Nutrition Examination Survey (NHANES) 2011–2016 demonstrated a significant association between serum copper levels and the risk of an elevated urinary albumin-to-creatinine ratio (ACR), a key marker of CKD progression (15). However, findings on the relationship between impaired renal function and serum copper levels are inconsistent. A study involving 68 CKD patients found no significant increase in serum copper or ceruloplasmin levels despite declining kidney function (16).

Mendelian randomization (MR) is a relatively novel causal inference method that utilizes genetic variants as instrumental variables, reducing the likelihood of bias from confounding effects compared to traditional observational studies. An MR study based on meta-analyses suggested that elevated copper levels might be a potential risk factor for CKD (17). Conversely, another MR study examined the relationship between trace elements, including copper, and various forms of CKD, such as hypertensive renal disease, diabetic nephropathy, IgA nephropathy, membranous nephropathy, and cystic nephropathy, and concluded that there was no causal link between copper levels and these kidney diseases (18). These findings have sparked interest in further investigating the association between copper, CKD, and renal fibrosis. They also emphasize the need for comprehensive research to elucidate the role of copper in CKD pathogenesis and progression.

To address this knowledge gap, we conducted MR analysis using data from genome-wide association studies (GWAS) and the FinnGen databases to explore the potential causal relationship between copper and CKD. Additionally, we validated this relationship in a retrospective cohort by examining serum copper levels and their association with CKD and renal fibrosis. The results of this research contribute to a deeper understanding of copper’s role in CKD progression and the development of renal fibrosis.

## 2 Materials and methods

### 2.1 MR study

MR is a robust analytical approach used to evaluate causal relationships between modifiable exposures or risk factors and clinically relevant outcomes. It is particularly valuable in scenarios where randomized controlled trials are impractical and where observational studies may be confounded by bias or reverse causality (19, 20). In this study, five MR methods, including MR-Egger, inverse-variance weighted (IVW), weighted mode, weighted median, and simple mode, were applied to investigate the causal relationship between serum copper levels and CKD.

The analysis utilized publicly available data from GWAS and the FinnGen database, which did not necessitate additional ethical approval. Serum copper levels were designated as the exposure variable, and CKD served as the outcome variable. Instrumental variables were selected based on stringent criteria: a *p*-value threshold of <5 × 10^−6^, minor allele frequency (MAF) > 1%, and a linkage disequilibrium (LD) threshold of R^2^ < 0.1 within a 5,000 kb window.

### 2.2 Study population

A retrospective study was conducted to validate the association between serum copper levels and CKD. The study included 505 CKD patients without dialysis admitted to the Department of Nephrology at Sichuan Provincial People’s Hospital between January 2021 and December 2022, along with 50 healthy volunteers serving as controls. The inclusion criteria were: (1) participants aged >18 years and (2) availability of trace element examination results. CKD diagnosis was based on the Kidney Disease Improving Global Outcomes (KDIGO) guidelines, defined as an eGFR <60 ml/min/1.73 m^2^ or a urine ACR ≥ 30 mg/g. CKD was staged into five categories according to KDIGO criteria (21). Exclusion criteria included pregnancy, serious infections, systemic diseases, or conditions such as severe malnutrition, cirrhosis, tumors, severe cardiovascular diseases, and hematopoietic or digestive system disorders.

### 2.3 Data collection

Baseline data, including patient demographics (age, gender) and blood analysis parameters (Serum creatinine, blood urea nitrogen, hemoglobin, albumin, and eGFR), were obtained from electronic health records at the time of study enrollment. Serum levels of copper, selenium, zinc, phosphorus, ionized calcium and chromium were measured using inductively coupled plasma mass spectrometry (ICP-MS), a sensitive and precise method for trace element determination (22).

### 2.4 Assessment of renal fibrosis

Of the 505 CKD patients, 168 underwent renal biopsy under ultrasound guidance at the time of study enrollment. The kidney tissue samples were fixed in formalin and embedded in paraffin, and the degree of renal fibrosis was assessed using Masson’s trichrome staining (23). The assessment was independently performed by two experienced renal pathologists to ensure accuracy and consistency in grading the extent of fibrosis.

### 2.5 Statistical analysis

MR analyses was performed using the “TwoSampleMR” package in R version 4.3.1. Various MR methods were employed, including MR-Egger, weighted median, simple mode weighted pattern analysis, and IVW analysis. Continuous variables were expressed as mean ± standard deviation (SD). To explore potential non-linear dose–response relationships between continuous exposures (serum copper levels) and outcomes, the restricted cubic spline (RCS) function was applied. This method is particularly effective for modeling non-linear associations and testing the hypothesis of linearity before incorporating continuous variables into models with appropriate recoding (24). For categorical data, group differences were assessed using T-tests, ANOVA, and chi-square tests. Multivariate logistic regression models were constructed to examine the association between serum copper levels, CKD risk and renal fibrosis, adjusting for potential confounders. Statistical significance was set at α = 0.05.

## 3 Results

### 3.1 MR analysis from GWAS and FinnGen databases

**Figure 1:**
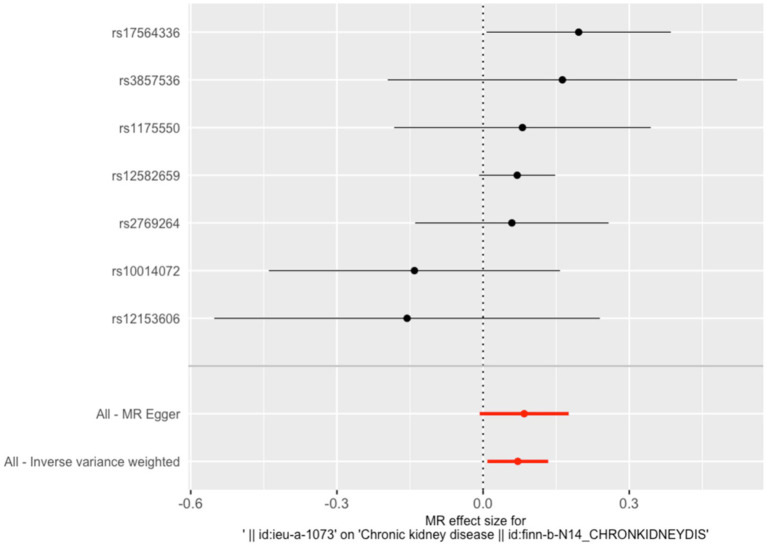
Effects of SNP on serum copper levels and CKD risk. Each data point on the plot corresponds to an instrumental variable single-nucleotide polymorphism (SNP). The lines extending from each point represent the 95% confidence intervals. The horizontal axis quantifies the effect of each SNP on the exposure factor, copper, while the vertical axis measures the impact of each SNP on the outcome, chronic kidney disease (CKD). The slope of the colored lines, differentiated by color to represent various algorithms, indicates the ratio of these two effects—reflecting the influence of copper on CKD. The consistent upward trajectory of these lines suggests a positive cor-relation between increased copper and a higher risk of CKD.

**Figure 2:**
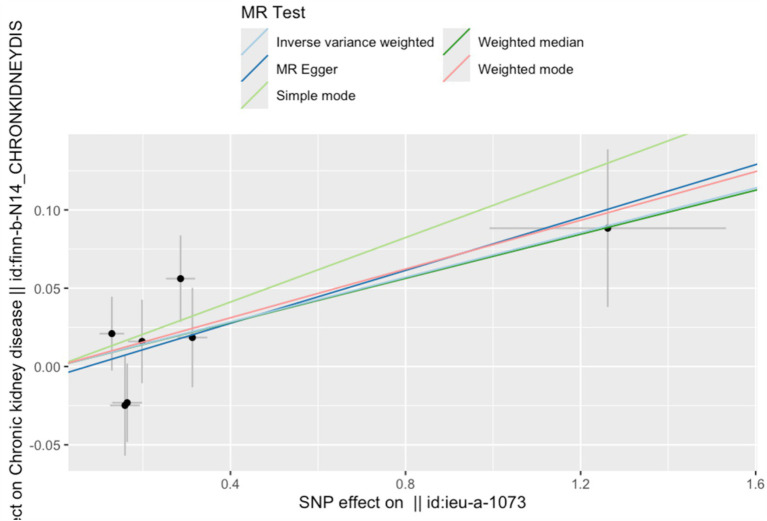
Forest plot of the association between serum copper levels and CKD risk. The vertical line situated at the center of the figure signifies the null line, indicating an odds ratio (OR) of 1. This indicates the absence of a statistical association between the studied factor and the outcome. Each horizontal line in the forest plot corresponds to the 95% confidence interval (CI) for a specific factor. The point at the center of each line represents the point estimate of the OR. If an entire horizontal line is positioned to the left of the null line, the factor is regarded as protective, suggesting a reduction in the incidence of the outcome (CKD). Conversely, if a horizontal line lies entirely to the right of the null line, the factor is deemed a risk factor, suggesting an increase in the incidence of the outcome.

The IVW results indicated that a one SD increase in genetically predicted serum copper levels was associated with a 5.8% higher risk of CKD (odds ratio (OR) = 1.058, 95% confidence interval (95% CI): 1.008–2.57, *p* = 0.005). Although the MR-Egger, weighted median, simple mode, and weighted mode analyses did not yield statistically significant results (*p* > 0.05), all methods showed a consistent positive association trend (all OR > 1; Table 1; Figure 1). The forest plot illustrates there was 1 SNP (rs17564336) associated with CKD, and the overall causal estimates derived from the IVW method (highlighted in red) suggested a potential causal relationship between the genetic variants and CKD (Figure 2). Furthermore, the leave-one-out analysis presented in the forest plot showed the error line is consistently positioned on right side of the x-axis at zero, indicating minimal overall variation (Supplementary Figure S1). Funnel plot illustrated the SNPs are approximately uniformly distributed around the IVW or MR Egger lines (Supplementary Figure S2). Taken together, the leave-one-out analysis supports that the MR results are robust.

**Table 1:** Impact of serum copper levels on CKD risk across five MR methods.

Exposure	Outcome	SNP	MR method	*p* value	OR (95%CI)
Copper	Chronic kidney disease	7	MR-Egger	0.129	1.050 (0.993–1.192)
		7	IVW	0.005	1.058 (1.008–1.429)
		7	Weighted mode	0.103	1.040 (0.998–1.117)
		7	Weighted median	0.139	1.047 (0.977–1.177)
		7	Simple mode	0162	1.046 (0.976–1.258)

CI, Confidence interval; IVW, Inverse-variance weighted; OR, Odds ratio; SNP, Single nucleotide polymorphism.

### 3.2 Serum copper levels in CKD patients

This study included 505 patients diagnosed with CKD and 50 healthy individuals serving as controls. Baseline characteristics revealed a mean age of 52.2 ± 17.3 years in the CKD group compared to 40.1 ± 13.6 years in the control group (*p* < 0.001). The proportion of males was comparable between the two groups, with 330 males (64.5%) in the CKD group and 34 males (68%) in the control group. CKD patients exhibited significantly elevated blood urea nitrogen (BUN) and serum creatinine (SCr) at 11.9 ± 8.5 mmol/L and 240.14 ± 250.64 μmol/L, respectively, compared to the control group (*p* < 0.001). Serum trace element analysis revealed significantly elevated copper levels and reduced selenium levels in CKD patients, while phosphorus, ionized calcium, and chromium levels showed no significant differences (Table 2).

**Table 2:** Baseline characteristics of CKD patients and healthy.

Before PSM				After PSM			
Characteristic	CKD (*n* = 505)	Healthy (*n* = 50)	*p* value	Characteristic	CKD (*n* = 100)	Healthy (*n* = 50)	*p* value
Age (years)	52.2 ± 17.3	40.1 ± 13.6	<0.001	Age (years)	40.2 ± 13.7	40.1 ± 13.6	0.983
Gender (male)	330 (64.5%)	34 (68.0%)	0.847	Gender (male)	67 (67.0%)	34 (68.0%)	0.902
BUN (mmol/L)	11.9 ± 8.5	5.2 ± 1.2	<0.001	BUN (mmol/L)	10.7 ± 7.1	5.2 ± 1.2	<0.001
SCr (μmol/L)	240.14 ± 250.64	79.80 ± 13.43	<0.001	SCr (μmol/L)	213.44 ± 209.99	79.80 ± 13.43	<0.001
Albumin (g/L)	34.6 ± 9.0	52.1 ± 17.2	<0.001	Albumin (g/L)	35.5 ± 8.9	52.1 ± 17.2	<0.001
Hb (g/L)	116.7 ± 30.5	150.2 ± 13.5	<0.001	Hb (g/L)	122.9 ± 26.2	150.2 ± 13.5	<0.001
Copper (μg/dl)	95.26 ± 26.62	79.55 ± 14.39	<0.001	Copper (μg/dl)	93.36 ± 27.19	79.55 ± 14.39	<0.001
Selenium (μg/dl)	7.64 ± 2.96	8.29 ± 1.85	0.014	Selenium (μg/dl)	7.46 ± 2.23	8.29 ± 1.85	0.012
Phosphorus (μg/dl)	142.53 ± 31.57	139.69 ± 36.31	0.55	Phosphorus (μg/dl)	142.55 ± 31.08	139.69 ± 36.31	0.616
Ionized calcium (μg/ml)	90.43 ± 10.10	89.49 ± 12.11	0.537	Ionized calcium (μg/ml)	90.93 ± 9.54	89.49 ± 12.11	0.427
Chromium (μg/dl)	1.03 ± 0.93	0.89 ± 0.42	0.605	Chromium (μg/dl)	0.98 ± 0.89	0.89 ± 0.42	0.408

CKD: Chronic kidney disease; PSM: propensity-score matching; BUN: Blood urea nitrogen, SCr: Serum creatinine, Hb: Hemoglobin.

To address the observed age, albumin (ALB) and hemoglobin (Hb) differences between the CKD and control groups, serum copper levels were re-evaluated after adjusting for age, ALB and Hb. Even after correction, serum copper remained significantly elevated in CKD patients (*p* < 0.05) (OR (95%CI) = 1.025(1.001, 1.050), *p* < 0.05) (Table 3). Propensity-score matching (PSM) was applied to minimize potential biases. PSM process adjusted age and gender between the 2 groups for final analysis. In PSM subgroup, CKD group had a significantly elevated levels of serum copper and reduced selenium levels (Table 2). Multivariate logistic regression analysis showed serum copper remained a risk factor for CKD (OR (95%CI) = 1.060(1.010, 1.121), *p* = 0.020) (Table 3).

**Table 3:** Multivariate logistic regression analysis of factors influencing serum copper levels in CKD.

Characteristic	CKD					
	Model 1		Model 2		Model 3	
	OR (95%CI)	*p* value	OR (95%CI)	*p* value	OR (95%CI)	*p* value
Serum copper	Before PSM					
	1.031 (1.016, 1.046)	<0.001	1.022 (1.007, 1.039)	0.005	1.025 (1.001, 1.050)	0.044
	After PSM					
	1.035 (1.013, 1.058)	0.002	1.037 (1.014, 1.060)	0.001	1.060 (1.010, 1.121)	0.020

Model 1: Unadjusted model. Model 2: Model was adjusted for age and gender. Model 3: Model was adjusted for age, ALB and Hb. CKD: Chronic kidney disease; PSM: propensity-score matching; ALB: Albumin; Hb: Hemoglobin. OR: Odds ratio; CI: Confidence interval.

### 3.3 Association of serum copper levels with CKD stages

The CKD cohort was stratified into five groups based on the KDIGO guidelines: CKD stage 1 (*n* = 121), stage 2 (*n* = 83), stage 3 (*n* = 107), stage 4 (*n* = 67), and stage 5 (*n* = 127). One-way ANOVA analysis revealed significant differences in serum levels of copper and selenium across the CKD stages (*p* < 0.05). Notably, serum copper demonstrated a progressive increase with worsening renal function, as indicated by advancing CKD stages. Conversely, selenium levels exhibited a declining trend as renal function deteriorated (Table 4). Correlation analysis showed that a positive association between serum copper levels and CKD stages (r = 1.000, *p* = 0.0167), while no correlation between serum selenium levels and CKD stages (r = −0.9000, *p* = 0.0833) (Supplementary Figure S3). Multivariate logistic regression analysis, adjusted for age, gender, albumin, and hemoglobin, revealed that serum copper levels were independently associated higher CKD stages, particularly in CKD 4 and 5 stage (OR (95%CI) = 1.021 (1.006, 1.037), *p* = 0.005; OR (95%CI) = 1.025 (1.010, 1.039), *p* < 0.001) (Table 5). These findings suggest a potential pathogenic role of serum copper in CKD progression, warranting further investigation into its mechanistic implications.

**Table 4:** Baseline characteristics across different CKD stages.

Characteristics	CKD G1 (*n* = 121)	CKD G2 (*n* = 83)	CKD G3 (*n* = 107)	CKD G4 (*n* = 67)	CKD G5 (*n* = 127)	*p* value
Age (years)	40.2 ± 15.1	51.4 ± 15.6	54.6 ± 17.1	60.0 ± 16.4	58.5 ± 15.0	<0.001
Gender (male)	66 (53.2%)	62 (73.8%)	78 (71.6%)	39 (56.5%)	99 (76.7%)	0.02
BUN (mmol/L)	5.3 ± 2.0	6.9 ± 2.2	9.2 ± 2.9	14.0 ± 4.5	22.9 ± 9.7	<0.001
SCr (μmol/L)	64.55 ± 14.37	91.77 ± 21.36	140.12 ± 41.52	232.74 ± 66.64	615.88 ± 261.82	<0.001
Albumin (g/L)	32.8 ± 9.4	34.4 ± 12.1	35.4 ± 8.1	35.7 ± 8.5	34.7 ± 7.4	<0.001
Hb (g/L)	134.9 ± 32.7	131.2 ± 19.5	121.3 ± 23.7	108.2 ± 19.8	90.4 ± 23.9	<0.001
Copper (μg/dl)	84.30 ± 22.42	88.66 ± 21.08	94.03 ± 24.48^*^	103.65 ± 26.52^*^	106.61 ± 29.67^*^	<0.001
Selenium (μg/dl)	8.02 ± 2.24	8.46 ± 4.41	7.97 ± 2.36	7.27 ± 2.39	6.66 ± 2.53^*^	<0.001

CKD: Chronic kidney disease; BUN: Blood urea nitrogen, SCr: Serum creatinine, Hb: Hemoglobin.

* Comparison with CKD G1 group, *p* < 0.05.

**Table 5:** Multivariate logistic regression analysis of factors influencing serum copper levels in CKD Stages.

CKD stage	Model 1		Model 2		Model 3	
	OR (95%CI)	*p* value	OR (95%CI)	*p* value	OR (95%CI)	*p* value
1	Ref.		Ref.		Ref.	
2	1.010 (0.996, 1.023)	0.152	1.003 (0.989, 1.017)	0.654	1.002 (0.988, 1.017)	0.763
3	1.020 (1.008, 1.032)	0.001	1.012 (0.999, 1.025)	0.072	1.011 (0.998, 1.025)	0.072
4	1.035 (1.022, 1.049)	<0.001	1.022 (1.008, 1.036)	0.002	1.021 (1.006, 1.037)	0.005
5	1.039 (1.027, 1.051)	<0.001	1.029 (1.016, 1.042)	<0.001	1.025 (1.010, 1.039)	<0.001

Model 1: Unadjusted model. Model 2: Model was adjusted for age and gender. Model 3: Model was adjusted for age, gender, ALB and Hb. CKD: Chronic kidney disease; ALB: Albumin; Hb: Hemoglobin. OR: Odds ratio; CI: Confidence interval.

### 3.4 Association of serum copper levels with renal fibrosis in CKD patients

Of the 168 CKD patients who underwent renal biopsy were included in this analysis. The degree of renal fibrosis was evaluated using Masson staining and categorized into two groups: Group 1 (fibrosis ≤ 5%, *n* = 114) and Group 2 (fibrosis > 5%, *n* = 54). Baseline characteristics, including age, gender, and albumin levels, showed no significant differences between the two groups. However, patients in Group 2 (higher fibrosis) exhibited significantly elevated serum creatinine and blood urea nitrogen levels, lower eGFR, and reduced hemoglobin levels compared to Group 1, consistent with advanced CKD clinical manifestations. Importantly, these patients also had significantly higher serum copper levels (Table 6).

**Table 6:** Association of serum copper levels with renal fibrosis severity in CKD patients.

Characteristics	Degree of fibrosis		
	Group 1 (≤5%) (*n* = 114)	Group 2 (>5%) (*n* = 54)	*p* value
Gender (male)	71 (62.3%)	40 (74.7%)	0.132
Age (years)	46.0 ± 16.23	48.82 ± 13.43	0.240
BUN (mmol/L)	6.7 ± 3.0	9.6 ± 4.4	< 0.001
SCr (μmol/L)	89.75 ± 38.27	150.81 ± 85.44	< 0.001
eGFR (ml/min/1.73m^2^)	84.40 ± 29.63	54.04 ± 24.11	< 0.001
Albumin (g/L)	33.1 ± 11.4	34.4 ± 9.4	0.462
Hb (g/L)	129.5 ± 29.8	120.2 ± 24.0	0.049
Copper (μg/dl)	83.10 ± 20.74	91.89 ± 23.98	0.016
Copper (μg/dl)^#^			
Q1 (<734.22)	33	9	0.003
Q2 (734.22–865.98)	22	20	
Q3 (865.99–961.64)	35	7	
Q4 (>961.64)	24	18	

BUN: Blood urea nitrogen, SCr: Serum creatinine, Hb: Hemoglobin, eGFR: Estimated glomerular filtration rate.

# Categorization of the serum copper levels into quartiles.

To further explore the relationship between serum copper levels and renal fibrosis, patients were stratified into quartiles based on serum copper concentration. Multivariate logistic regression analysis, adjusted for age, gender, albumin, and hemoglobin, revealed that higher serum copper levels (Quartile 4: > 96.16 μg/dl) were independently associated with higher renal fibrosis (OR = 2.75, 95% CI = 1.06–7.16, *p* < 0.001; Table 7). Additionally, RCS analysis demonstrated a non-linear relationship between serum copper levels, eGFR, and the degree of renal fibrosis (P_nonlinear_ = 0.038 and 0.005, respectively; Supplementary Figures S4, S5). These findings further demonstrated that elevated serum copper levels may play a role in the progression of renal fibrosis in CKD patients.

**Table 7:** Logistic regression analysis of the association between serum copper levels and renal fibrosis in CKD patients.

Characteristics	Degree of renal fibrosis (≥ 5%)					
	Model 1		Model 2		Model 3	
	OR (95%CI)	*p* value	OR (95%CI)	*p* value	OR (95%CI)	*p* value
Serum copper						
Q1	Ref		Ref		Ref	
Q2	3.33 (1.28, 8.65)	0.013	3.27 (1.24, 8.62)	0.016	3.94 (1.42, 10.97)	0.009
Q3	0.73 (0.25, 2.20)	0.579	0.79 (0.26, 2.42)	0.682	0.82 (0.26, 2.60)	0.736
Q4	2.75 (1.06, 7.16)	<0.001	2.91 (1.07, 7.90)	0.036	3.11 (1.11, 8.74)	0.031
*p* for trend^#^		0.020		0.012		0.010

Model 1: Unadjusted model. Model 2: Model was adjusted for age and gender. Model 3: Model was adjusted for age, gender, ALB and Hb. ALB: Albumin; Hb: Hemoglobin. OR: Odds ratio; CI: Confidence interval.

# Test for trend based on continuous variable.

## 4 Discussion

In the present study, we established a causal relationship between serum copper levels and CKD from GWAS and FinnGen database via MR analysis. And our real-world retrospective study further demonstrated that elevated serum copper levels are a significant risk factor for CKD, showing a positive correlation with disease severity across CKD stages. Furthermore, we investigated the association between serum copper and renal fibrosis in biopsy-proven CKD patients, revealing that higher serum copper levels were independently associated with increased renal fibrosis, thereby underscoring the potential role of copper metabolism in fibrosis pathogenesis.

MR is a powerful approach that employs genetic variation as an instrumental variable to assess causal relationships, allowing for better control of confounding factors and reverse causality. This method thus facilitates a more accurate evaluation of the causal effects of exposures on outcome (25). So, this approach allows for a more precise evaluation of the causal effects of serum copper on CKD outcomes. MR has been recognized as a valuable tool in kidney disease research (26). Our findings align with the study conducted by Ahmad et al. (17), which also identified a potential genetic causal relationship between elevated copper levels and CKD. However, unlike our study, Ahmad et al. relied solely on genomic data from a meta-analysis of three cohorts, whereas we integrated genetic data with real-world clinical observations to enhance the robustness of our conclusions.

In our retrospective cohort analysis, we analysis baseline demographic data and serum trace element levels results from 505 CKD patients and 50 healthy controls. Our results revealed that serum copper levels were significantly elevated in CKD patients compared to the healthy individuals, corroborating previous studies conducted in older adult Chinese populations and rural communities in Bangladesh (13, 27). These findings suggest that elevated serum copper levels may serve as a potential risk factor for CKD. Moreover, we observed a progressive increase in serum copper levels as CKD advanced from stage 1 to stage 5, reinforcing the hypothesis that copper may play a critical role in CKD progression. Compared to previous studies (10, 12, 28–30) that focused on specific populations such as older adult individuals, occupational cohorts, or dialysis patients, this study is a retrospective study of patients with CKD and the inclusion of healthy controls, which allowed for a more robust analysis of the relationship between serum copper levels and CKD staging. The combination of existing researches and our results strengthens the case for copper may as a contributing factor to CKD progression, however, the mechanism of serum copper causes CKD is still unclear, which needs to be explored by further studies.

Renal fibrosis is a well-known pathological feature of various forms of CKD and plays a significant role in the progression of CKD. In the present study, we investigated the relationship the relationship between serum copper levels and renal fibrosis in CKD patients. Our findings revealed a significant association between elevated serum copper levels and the severity of renal fibrosis, underscoring the importance of copper metabolism in fibrotic pathogenesis.

The mechanisms underlying copper-induced renal fibrosis are complex and multifaceted. Previous research has shown that copper ions can stimulate fibroblast proliferation and promote their differentiation into myofibroblasts, which are characterized by increased expression of alpha-smooth muscle actin (α-SMA) and enhanced extracellular matrix (ECM) production (31). Additionally, both inflammation and oxidative stress play critical roles in the development of renal fibrosis, and dysregulated of copper homeostasis can induce oxidative stress (7). Excessive copper accumulation has been shown to contribute to mitochondrial dysfunction and oxidative stress, further accelerating the progression of renal fibrosis (32).

Recent studies have also highlighted the role of cuproptosis, a form of copper-induced cell death, in kidney injury (33). In both *in vitro* and *in vivo* nephropathy models, interventions targeting copper overload have demonstrated protective effects by attenuating cuproptosis and mitigating renal damage (9, 34). Although increased copper ion concentrations can lead to varying degrees of cellular damage, it is noteworthy that moderate copper-induced cell death has been shown to alleviate pulmonary fibrosis by inhibiting myofibroblast activation (31). In our study, we identified a non-linear relationship between serum copper levels and renal fibrosis. These findings reinforce the need for further mechanistic studies to explore the precise role of copper in fibrosis progression.

Furthermore, studies in animal models have demonstrated that copper chelation therapy can mitigate renal fibrosis. In a unilateral ureteral obstruction (UUO) rat model, elevated copper was observed within renal tissues. Treatment with a copper chelator, tetrathiomolybdate, significantly reduced copper concentrations in renal tissues and improved renal fibrosis (35). Similarly, treatment with tetrathiomolybdate could also alleviate renal fibrosis in UUO mice (36). Interestingly, research on tetrathiomolybdate therapy in pulmonary fibrosis models has shown promising results, reducing fibrosis severity and vascular density (37). These findings suggest that modulating copper metabolism could represent a novel therapeutic avenue for CKD-related fibrosis, warranting further clinical investigations.

This study is, to the best of our knowledge, this study is the first systematic investigation to examine the relationship between serum copper levels, CKD, and renal fibrosis using a combination of MR and retrospective cohort analysis. However, some limitations should be acknowledged. First, the single-center nature of our retrospective study may impact the generalizability of our findings. Second, the small-size of the patient sample may not be representative, multicenter validation studies are necessary to confirm these results. Additionally, while MR analysis strengthens causal inference, it relies on the assumption that genetic variants influence CKD exclusively through serum copper levels. Potential pleiotropic effects may introduce bias, necessitating further investigation. Finally, future *in vivo* and *in vitro* studies are required to uncover the precise molecular mechanisms linking copper metabolism to renal fibrosis.

## 5 Conclusion

Our findings suggest that elevated serum copper levels may serve as a causal risk factor for CKD and renal fibrosis. These results highlight the importance of monitoring copper levels in CKD patients and underscore the need for therapeutic strategies targeting copper metabolism to mitigate fibrosis and improve kidney health. Future studies should focus on clinical trials assessing copper chelation therapy and dietary interventions as potential approaches to managing CKD-associated fibrosis.

## Data Availability

The original contributions presented in the study are included in the article/Supplementary material, further inquiries can be directed to the corresponding author.
