# Supplementary figures and images for: Association between copper exposure and renal fibrosis in patients with chronic kidney disease: evidence from Mendelian randomization and a retrospective study
Source: Front Public Health. 2025 Aug 25;13:1657180. doi: 10.3389/fpubh.2025.1657180 (PMC12414938; doi:10.3389/fpubh.2025.1657180)

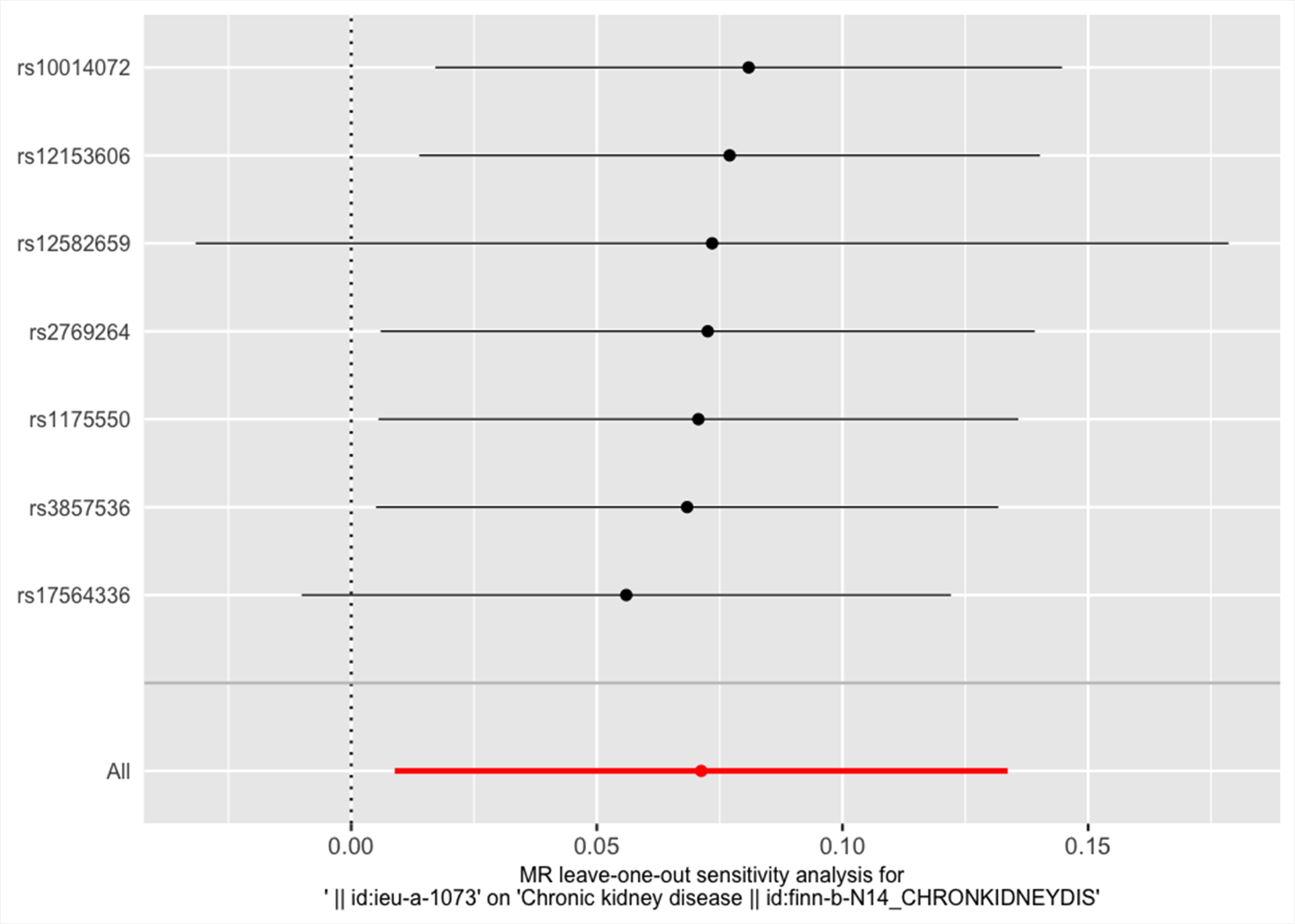

Supplement: Supplementary file 1 [file Image_1.tiff]

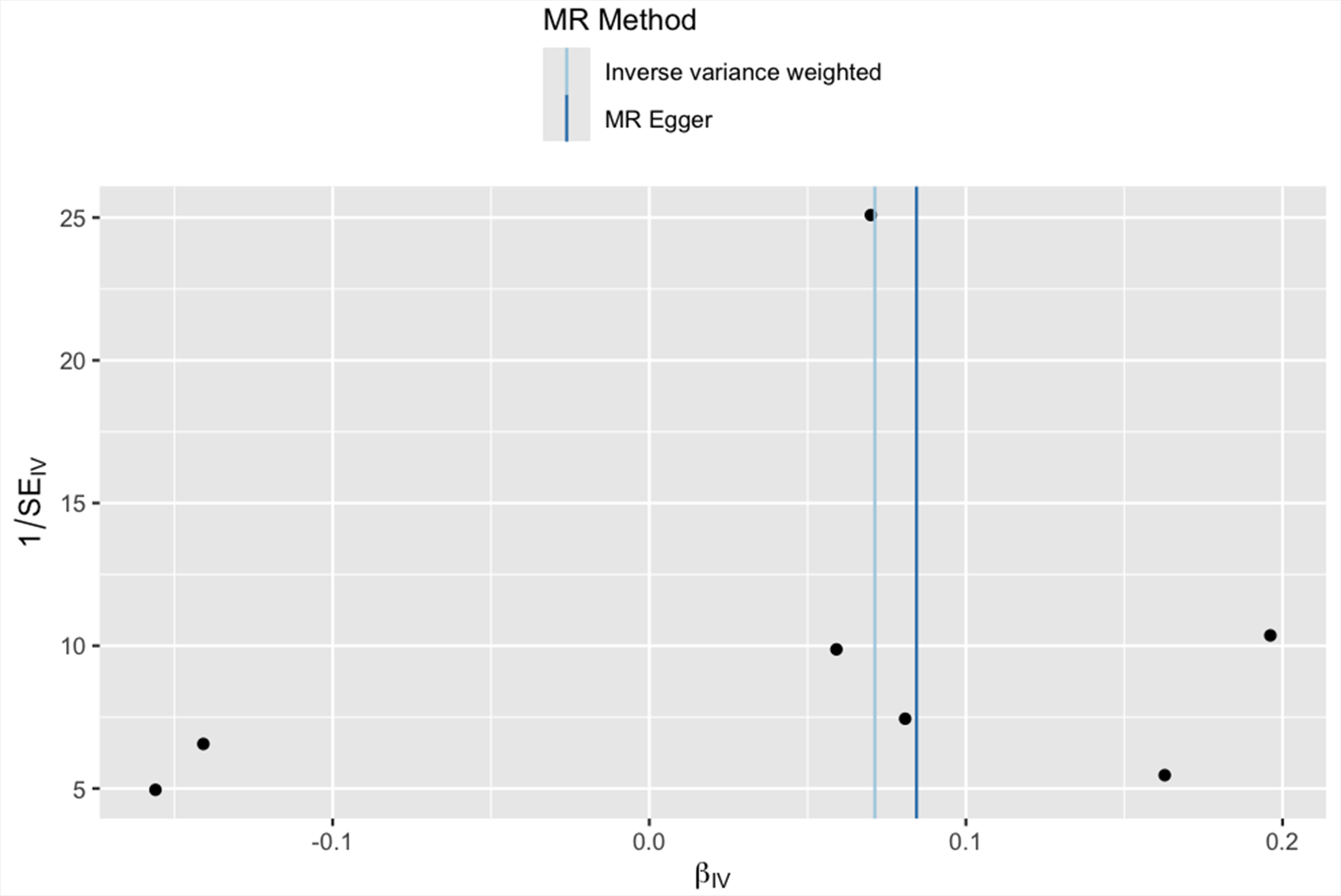

Supplement: Supplementary file 2 [file Image_2.tiff]

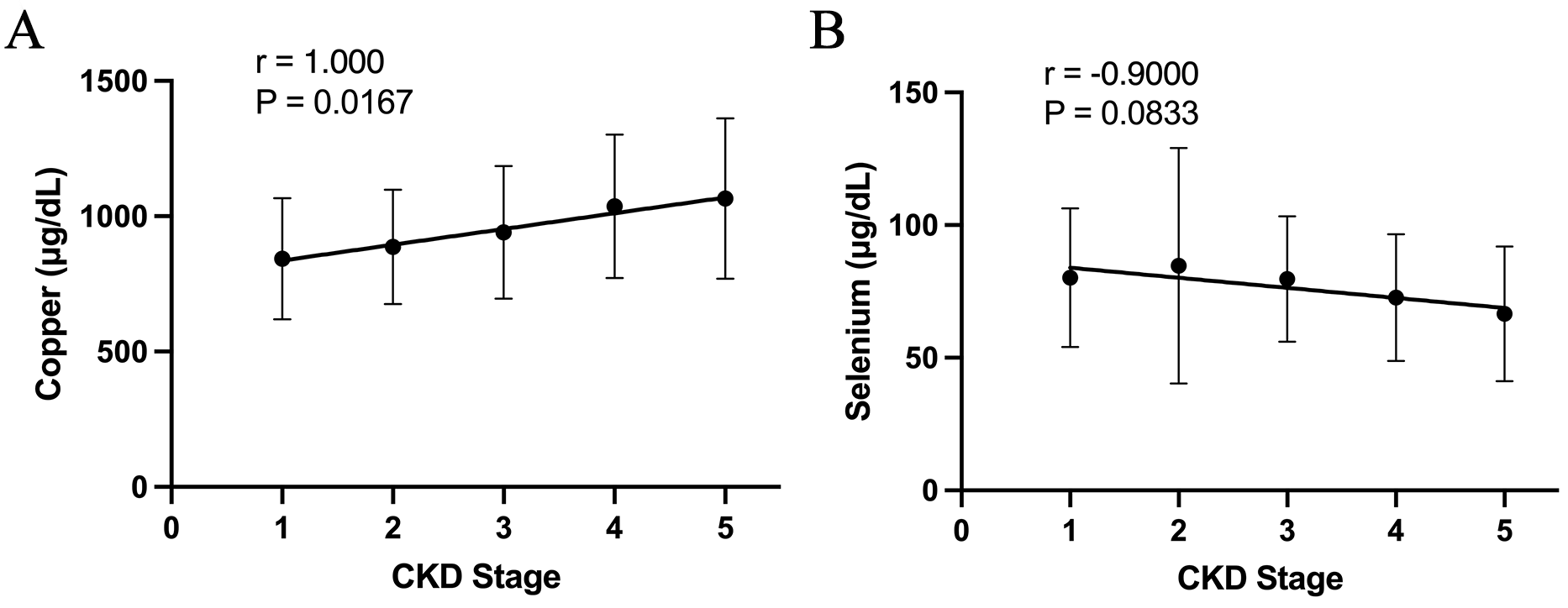

Supplement: Supplementary file 3 [file Image_3.tiff]

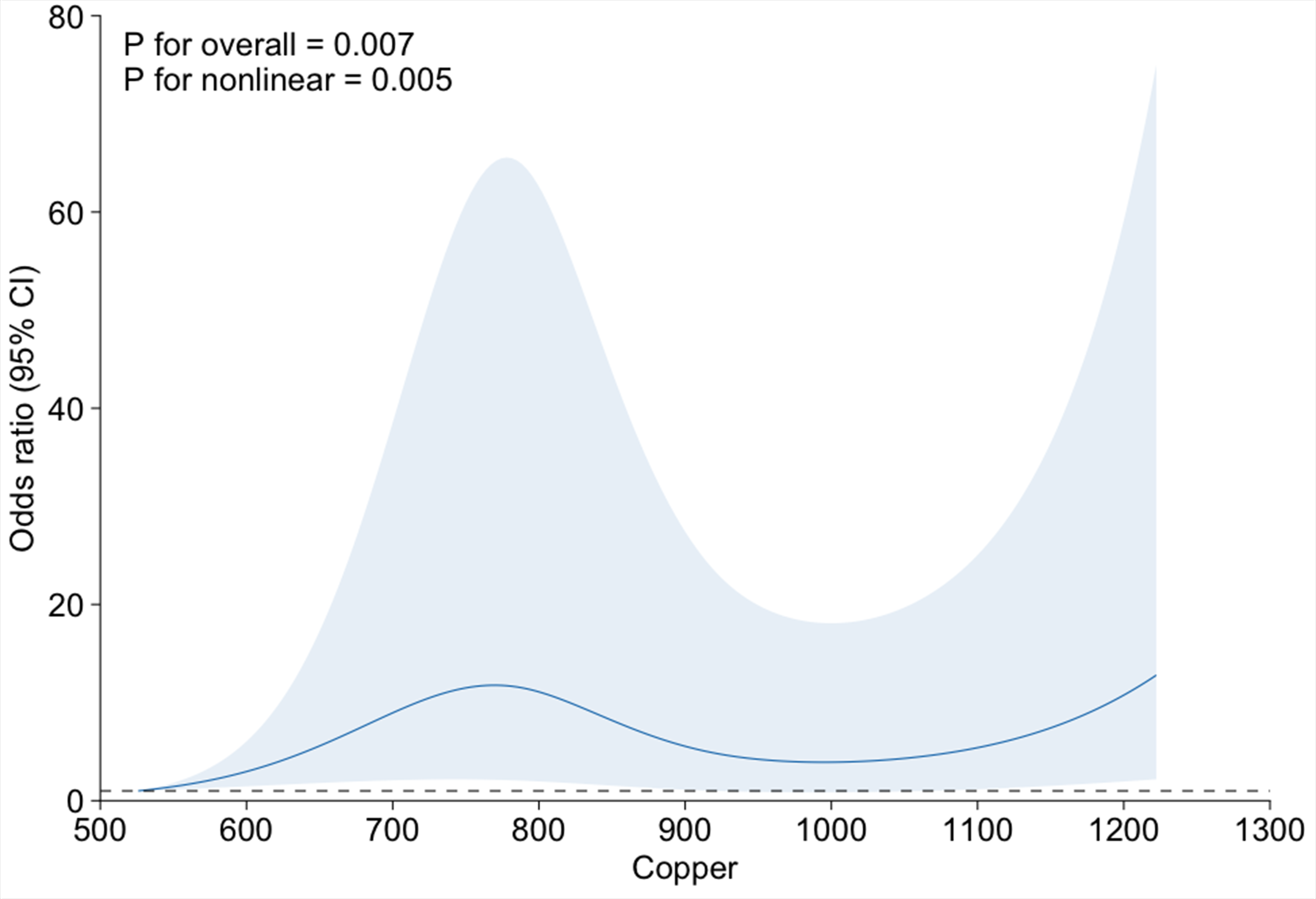

Supplement: Supplementary file 4 [file Image_4.tif]

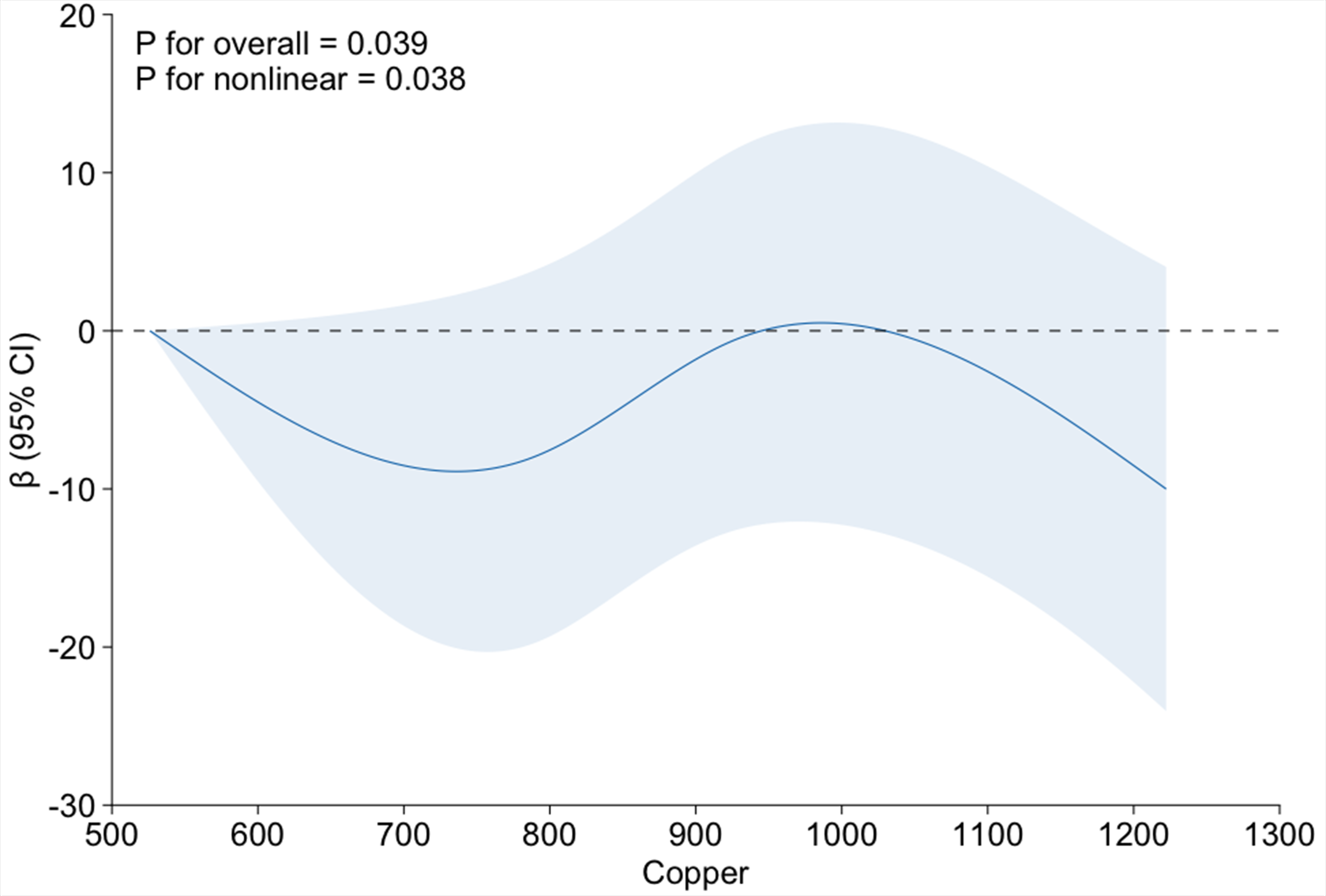

Supplement: Supplementary file 5 [file Image_5.tif]
